# A neural mass model of cross frequency coupling

**DOI:** 10.1371/journal.pone.0173776

**Published:** 2017-04-05

**Authors:** Mojtaba Chehelcheraghi, Cees van Leeuwen, Erik Steur, Chie Nakatani

**Affiliations:** 1 Brain and Cognition Research Unit, KU Leuven, Leuven, Belgium; 2 Center for Cognitive Science, TU Kaiserslautern, Kaiserslautern, Germany; Georgia State University, UNITED STATES

## Abstract

Electrophysiological signals of cortical activity show a range of possible frequency and amplitude modulations, both within and across regions, collectively known as cross-frequency coupling. To investigate whether these modulations could be considered as manifestations of the same underlying mechanism, we developed a neural mass model. The model provides five out of the theoretically proposed six different coupling types. Within model components, slow and fast activity engage in phase-frequency coupling in conditions of low ambient noise level and with high noise level engage in phase-amplitude coupling. Between model components, these couplings can be coordinated via slow activity, giving rise to more complex modulations. The model, thus, provides a coherent account of cross-frequency coupling, both within and between components, with which regional and cross-regional frequency and amplitude modulations could be addressed.

## Introduction

Electrophysiological activity of interconnected neurons encompasses multiple oscillatory components [1,2]; these are subject to modulation of frequency [3–5] and amplitude [6,7]. Modulation may have various utilities [8,9], such as sequence encoding [10,11], rectification of local neural activity [12,13] and long-rage information transfer [14,15]. The mechanisms behind these effects, however, are still not well-understood. In theory, they could all be manifestations of a single underlying principle. To contemplate this possibility, we propose a model that describes frequency and amplitude modulations as systematic relationships between slow and fast oscillatory components of neural population activity.

Systematic relationships between oscillatory components are collectively known as cross-frequency coupling (CFC). Jensen & Colgin [2] listed six types of CFC of interest to electrophysiology: phase-phase, phase-frequency, phase-amplitude, frequency-frequency, amplitude-amplitude and amplitude-frequency couplings (PPC, PFC, PAC, FFC, AAC, and AFC, respectively). Empirical studies have mostly observed PPC [16–20], PAC [21–23] and occasionally AAC [24–26]. To our knowledge, PFC, AFC and FFC have not been empirically observed so far. Shortcomings of current CFC detection methods may, in part, be responsible for this. In particular, PFC may have been misidentified as PAC [9,27]. In anticipation of further progress in measurement, it is desirable to have a model predicting the conditions under which these modulations could arise, as part of an encompassing account of CFC.

We propose a neural mass model (NMM) for CFC. The neural mass approach collectively describes neuronal activity by representing it at the level of neural populations. NMMs generate oscillatory activities through the interaction of neuronal populations within and across regions. NMMs have been successfully used to describe various mesoscopic brain activities [28] such as ongoing alpha, beta, gamma, delta, and theta band activity [29–31], the sleep spindle/k-complex [32,33], as well as evoked activity [34,35].

Recently we introduced a NMM that manifests PAC [36]. The model consists of four neural populations: pyramidal neurons, excitatory interneurons, slow and fast inhibitory interneurons. A noise source, which represents un-modeled activity of neighboring brain regions, serves as external input. The noise drives both the pyramidal neuron and the fast inhibitory interneuron populations. Like its predecessor NMMs, the four-populations model generates slow activity comparable to alpha, theta, and delta band oscillations through interactions between pyramidal neuron and slow-inhibitory interneuron populations. Unique to our NMM, the fast inhibitory interneuron population has a dynamic self-feedback mechanism, which plays a crucial role in generating fast oscillatory activity.

Within this mechanism, the mean level of noise input acts as a bifurcation parameter for the fast inhibitory interneuron population. For zero noise level, no fast oscillations arise. As the noise level is increased, fast activity emerges in the model’s output, due to the self-feedback of the fast-inhibitory interneuron population. The fast activity is comparable to interneuron gamma (ING), which has been observed in neocortex and allocortex, e.g., in visual cortices and hippocampus [37–39]. In Chehelcheraghi et al. [36], we demonstrated that the mean level of input noise is a critical factor for the modulation of fast activity (see also [40]). From a certain nonzero noise level, fast oscillations occur and above a critical level, the model produces a PAC signal: modulation of fast activity *amplitude* by the phase of the slow rhythm. Here we will first show that noise below that level leads to PFC: modulation of fast activity *frequency* by the phase of the slow rhythm. Next, we will demonstrate that besides PAC and PFC, other types of CFCs, namely FFC, AAC and AFC can all be modeled based on the four-populations model, depending solely on the choice of mean input noise level.

The four-populations model is considered to represent a cortical patch between a few millimeters and a centimeter in diameter, in which oscillations in various frequencies can be observed [41]. Thus, we considered the four-populations model to represent a local region. Empirical studies reports CFC between local regions. For instance, AAC was observed across brain regions such as superior temporal and fusiform gyri [42]. We therefore connected two identical four-populations models symmetrically via populations of pyramidal neurons. These between-region populations relay slow oscillatory activity from one region to the other, and allow the slow oscillations within both components to become synchronized. Synchronized slow activity thus serves as a common modulator for the fast activities within both regions. The between-region pyramidal populations could, in principle, be replaced by or extended with an arbitrary oscillator (e.g., a thalamic pacemaker [43]). While allowing such possible extensions, the current study used pyramidal units which we consider as the generic between-region connectivity.

Although both PFC and PAC can arise within a single component, here we will operate from the onset with a two components of the four-populations model, to show the role of synchronization of the slow activity between the components in modeling cross-regional PAC and PFC. In the first simulation, the two components of the four-populations model were used to show PFC across the regions. Similarly, cross-regional PAC was shown in the second simulation. In both simulations, the second component has zero mean noise level, thereby preventing any fast oscillations to emerge within the second component. Thus, cross regional PAC and PFC arise via synchronization with the slow oscillations that connect the two components. In the third, fourth, and fifth simulations, FFC, AAC and AFC were generated, respectively, each as a function of the levels of nonzero mean noise input to both components. Based on the results, we propose the present model as an inclusive theoretical framework for the different CFC phenomena.

## Methods

### Overview

**Fig 1:**
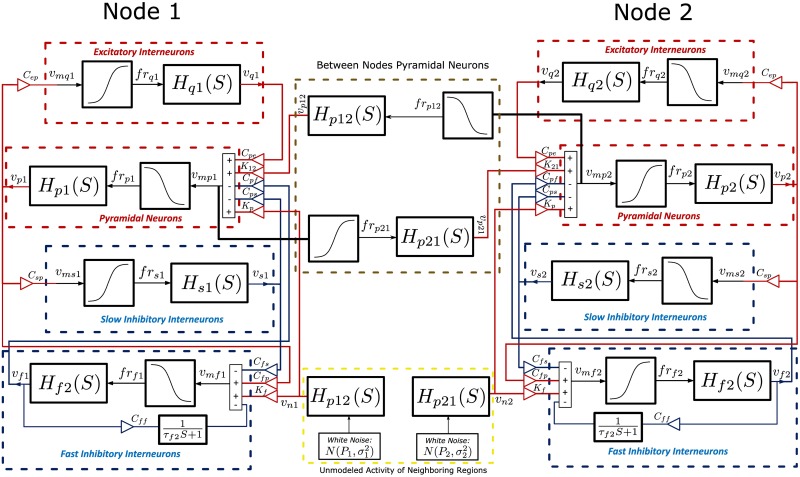
Schematic diagram of the proposed model. Each node is comprised of four neural population units: pyramidal neurons, excitatory interneurons, slow inhibtory interneurons and fast ihbitory internneurons. Each node is identical to the NMM in [36]. The nodes are mutually connected by the dynamics of the pyramidal neurons. Two independent Gaussian noise sources drive the NMM nodes.

As illustrated in Fig 1, the present model consists of two identical components labelled as Node 1 and Node 2. Each component is a four-populations NMM node which simulates electrophysiological activity [36]. The four populations represent, respectively, pyramidal neuron, excitatory interneuron, slow inhibitory interneuron, and fast inhibitory interneuron of the cortex. In each node, interaction between the pyramidal neurons and slow inhibitory interneurons generates a steady-state activity, which is considered as its main (intrinsic) oscillation (e.g. alpha band activity in visual cortex in [33]. Unique to our four-populations model, the fast-inhibitory interneuron population has dynamic self-feedback. The feedback enables the fast inhibitory interneurons to generate oscillations in the gamma range. This fast activity can occur simultaneously with the slow activity, which is a prerequisite for CFC in the model. The two four-populations nodes are mutually connected via two between-node pyramidal neuron populations.

The emergence and stability of fast activity in each node depends on two parameters, *P*_*1*_ and *P*_*2*_, which represent the mean levels of noise input to the nodes [36]. Small values deactivate the fast oscillations (damping). Values above the damping regime determine the type of modulation between the slow and fast oscillation. For parameter values below a critical threshold, resonance occurs, allowing frequency modulation, namely, PFC, AFC, and FFC; above the threshold the fast oscillations reach a steady-state, in which the frequency is invariant, and amplitude modulation results, allowing for PAC and AAC. For an analytical account of this mechanism, see [44]). We systematically vary the values of *P*_*1*_ and *P*_*2*_ in order to demonstrate that PFC, PAC, FFC, AAC and AFC appear as a function of the parameters *P*_*1*_ and *P*_*2*_, without changing model structure or connectivity.

### Model

Each of the four neural population units of a node, pyramidal neurons, excitatory interneurons, slow inhibitory interneurons, and fast inhibitory interneurons, has two variables: mean firing rate *fr*(*t*) which is a function of mean membrane potential *v*_*m*_(*t*), and mean post synaptic potential *v*(*t*). The mean membrane potential is obtained from the weighted summation of inputs. The mean membrane potential is converted to the mean firing rate via a sigmoid function:

$$fr(t)=Sig\left(v_{m}\left(t\right)\right)=\frac{\upsilon_{\text{max}}}{1+\text{exp}(-r\left(v_{m}\left(t\right)-v_{\theta }\right))}$$

Here *v*_max_ is the maximum firing rate of the population of neurons, *v*_*θ*_ is the value of the potential for which a 50% mean firing rate is achieved, and r is the slope of the sigmoid at *v*_*θ*_; The parameter *v*_*θ*_ will serve as mean firing threshold. The sigmoid function is parametrized equally for all populations in the model. A second-order differential equation called the dendritic transfer function relates the mean firing rate, *fr*(*t*), of a population to the un-weighted mean post synaptic potential, *v*(*t*), of that population via the dendritic transfer function:

$$H(S)=\frac{G\omega }{S^{2}+2\omega S+\omega^{2}},$$

where *G* and *ω* characterize the mean strength and speed, respectively. Different population units are distinguished by their time constant $T=\frac{1}{\omega }\;\left[sec\right]$ and the low frequency gain of the dendritic transfer function $\frac{G}{\omega }\;[mV.\text{sec}]$. The dendritic transfer function of each population unit is denoted as *H*_*lx*_(*S*), in which l ∈ {p: pyramidal neuron population, q: excitatory interneuron population, f: fast inhibitory interneuron population, s: slow inhibitory interneuron population} and x ∈ {1,2} is the node number, e.g. *H*_*s*2_(*S*) denotes the dendritic transfer function of the slow inhibitory interneuron population in the second node.

Four interconnected population units constitute a single node. Inputs to each population unit are the weighted outputs from the other units, reflecting the mean excitatory or inhibitory post synaptic potentials of an excitatory or inhibitory population (EPSP or IPSP). The input synaptic potentials are weighted by constant connectivity strengths which are called synaptic gains. A synaptic gain between populations is represented by *C*_*αβ*_ where *α*, *β* ∈ {p: pyramidal neuron population, q: excitatory interneuron population, f: fast inhibitory interneuron population, s: slow inhibitory interneuron population}. Here *α* and *β* refer to target and source population, respectively; e.g. the constant *C*_*ps*_ is the gain of the synaptic connection from the slow inhibitory population to the pyramidal neuron population.

The pyramidal neuron population unit excites all three interneuron population units within the node and in turn receives input from them. The slow inhibitory interneuron population unit inhibits the fast inhibitory interneuron population unit. The fast inhibitory interneuron population has an extra state variable, *v*_*ff*_*(t)*, for the dynamic self-inhibition. The dynamics of the self-feedback in the fast inhibitory population is of first order and serves as a low pass filter with cut-off frequency $f_{c}=\frac{1}{\left(2\pi \tau_{f}\right)}$.

As shown in Fig 1, Nodes 1 and 2 have identical structure. The two nodes are mutually connected via the pyramidal populations. The synaptic gain of Node 1 to Node 2 is represented by *K*_21_, while that in the inverse direction is given as *K*_12_. The dendritic transfer functions of pyramidal neurons between the nodes are labeled as *H*_*p*21_(*S*) and *H*_*p*12_(*S*). For each node, independent Gaussian noise excites both the fast inhibitory interneuron and pyramidal neuron units. The noise represents the mean firing rate of unmodeled external neural populations. The noise is passed through the dendritic transfer function of pyramidal neurons between the nodes, i.e., *H*_*p*21_(*S*) and *H*_*p*12_(*S*), in order to convert it to mean post synaptic potentials. The potentials are weighted by factors *K*_*p*_ and *K*_*f*_ in order to excite the pyramidal neurons and fast inhibitory interneurons, respectively. For each node, the mean membrane potential of the pyramidal population is the main output. The pyramidal population unit has been the main output (and input) unit in previous cortical neural mass models [29,35]. The mean membrane potential of the neurons represents population activity of the node; thus, this output is comparable with macroscopic electrophysiological brain signals, such as, LFP, ECoG, and EEG. The dynamics of the full model is described as follows.

#### Equations for Node 1

Pyramidal neuron population:

$$\frac{d^{2}v_{p1}}{dt^{2}}=G_{p1}\omega_{p1}Sig\left(v_{mp1}\left(t\right)\right)-2\omega_{p1}\frac{dv_{p1}}{dt}-\omega_{p1}^{2}v_{p1}\left(t\right)$$

$$v_{mp1}\left(t\right)=C_{pq}v_{q1}\left(t\right)-C_{ps}v_{s1}\left(t\right)-C_{pf}v_{f1}\left(t\right)+K_{12}v_{p12}\left(t\right)+K_{p1}v_{n1}(t)$$

Excitatory interneuron population:

$$\frac{d^{2}v_{q1}}{dt^{2}}=G_{q1}\omega_{q1}Sig\left(v_{mq1}\left(t\right)\right)-2\omega_{q1}\frac{dv_{q1}}{dt}-\omega_{q1}^{2}v_{q1}\left(t\right)$$

$$v_{mq1}\left(t\right)=C_{qp}v_{p1}\left(t\right)$$

Slow inhibitory interneuron population:

$$\frac{d^{2}v_{s1}}{dt^{2}}=G_{s1}\omega_{s1}Sig\left(v_{ms1}\left(t\right)\right)-2\omega_{s1}\frac{dv_{s1}}{dt}-\omega_{s1}^{2}v_{s1}\left(t\right)$$

$$v_{ms1}\left(t\right)=C_{sp}v_{p1}\left(t\right)$$

Fast inhibitory interneuron population:

$$\frac{d^{2}v_{f1}}{dt^{2}}=G_{f1}\omega_{f1}Sig\left(v_{mf1}\left(t\right)\right)-2\omega_{f1}\frac{dv_{f1}}{dt}-\omega_{f1}^{2}v_{f1}\left(t\right)$$

$$\tau_{f1}\frac{dv_{ff1}\left(t\right)}{dt}=-v_{ff1}\left(t\right)+v_{f1}\left(t\right)$$

$$v_{mf1}\left(t\right)=C_{fp}v_{p1}\left(t\right)-C_{fs}\left(t\right)v_{s1}\left(t\right)-C_{ff}v_{ff1}\left(t\right)+K_{f1}v_{n1}(t)$$

External inputs:

$$\frac{d^{2}v_{p12}}{dt^{2}}=G_{p12}\omega_{p12}Sig(v_{mp2}(t))-2\omega_{p12}\frac{dv_{p12}}{dt}-\omega_{p12}^{2}v_{p12}\left(t\right)\;\left(From\;Node\;2\right)$$

$$\frac{d^{2}v_{n1}}{dt^{2}}=G_{p12}\omega_{p12}n_{1}(t)-2\omega_{p12}\frac{dv_{n1}}{dt}-\omega_{p12}^{2}v_{n1}\left(t\right)\;\left(Filtered\;noise\right)$$

$$n_{1}\left(t\right)=N\left(P_{1},\sigma_{1}^{2}\right)\;\left(White\;noise\right)$$

#### Equations for Node 2

Pyramidal neuron population:

$$\frac{d^{2}v_{p2}}{dt^{2}}=G_{p2}\omega_{p2}Sig\left(v_{mp2}\left(t\right)\right)-2\omega_{p2}\frac{dv_{p2}}{dt}-\omega_{p2}^{2}v_{p2}\left(t\right)$$

$$v_{mp2}\left(t\right)=C_{pq}v_{q2}\left(t\right)-C_{ps}v_{s2}\left(t\right)-C_{pf}v_{f2}\left(t\right)+K_{21}v_{p21}\left(t\right)+K_{p2}v_{n2}(t)$$

Excitatory interneuron population:

$$\frac{d^{2}v_{q2}}{dt^{2}}=\;G_{q2}\omega_{q2}Sig\left(v_{mq2}\left(t\right)\right)-\;2\omega_{q2}\frac{dv_{q2}}{dt}-\;\omega_{q2}^{2}v_{q2}\left(t\right)$$

$$v_{mq2}\left(t\right)=C_{qp}v_{p2}\left(t\right)$$

Slow inhibitory interneuron population:

$$\frac{d^{2}v_{s2}}{dt^{2}}=G_{s2}\omega_{s2}Sig\left(v_{ms2}\left(t\right)\right)-2\omega_{s2}\frac{dv_{s2}}{dt}-\omega_{s2}^{2}v_{s2}\left(t\right)$$

$$v_{ms2}\left(t\right)=C_{sp}v_{p2}\left(t\right)$$

Fast inhibitory interneuron population:

$$\frac{d^{2}v_{f2}}{dt^{2}}=G_{f2}\omega_{f2}Sig\left(v_{mf2}\left(t\right)\right)-2\omega_{f2}\frac{dv_{f2}}{dt}-\omega_{f2}^{2}v_{f2}\left(t\right)$$

$$\tau_{f2}\frac{dv_{ff2}\left(t\right)}{dt}=-v_{ff2}\left(t\right)+v_{f2}\left(t\right)$$

$$v_{mf2}\left(t\right)=C_{fp}v_{p2}\left(t\right)-C_{fs}\left(t\right)v_{s2}\left(t\right)-C_{ff}v_{ff2}\left(t\right)+K_{f2}v_{n2}(t)$$

External inputs:

$$\frac{d^{2}v_{p21}}{dt^{2}}=G_{p21}\omega_{p21}Sig(v_{mp1}(t))-2\omega_{p21}\frac{dv_{p21}}{dt}-\omega_{p21}^{2}v_{p21}\left(t\right)\;\left(From\;Node\;1\right)$$

$$\frac{d^{2}v_{n2}}{dt^{2}}=G_{p21}\omega_{p21}n_{2}(t)-2\omega_{p21}\frac{dv_{n2}}{dt}-\omega_{p21}^{2}v_{n2}\left(t\right)\;\left(Filtered\;noise\right)$$

$$n_{2}\left(t\right)=N\left(P_{2},\sigma_{2}^{2}\right)\;\left(White\;noise\right)$$

#### Parameters

Table 1 shows the parameter values that are common to all current CFC simulations. The two nodes are identically parametrized, except for the time constant of the dynamic self-feedbacks in the fast inhibitory interneurons, *τ*_*f*1_ and *τ*_*f*2_. As we will explain further in the subsection *Choice of Slow and Fast Oscillation Frequencies*, these parameters were fixed at different values for the sake of obtaining FFC, AAC and AFC. The remaining parameter values are those suggested by [33] and [30], same as in our previous study [36]. Jansen and Rit [33] assumed that synaptic connections are active in equal proportions between interacting units, and proposed to the fix ratios of synaptic gains. The set of parameters was modified by Wendling et al., [30] to model fast inhibitory interneurons. The modified set of parameters has been the basis set for fast oscillations in many neural mass modeling studies, including the current one.

**Table 1:** Model parameters: Fixed values.

Parameter Interpretation	Notation	Value
Synaptic gain from excitatory interneurons to pyramidal neurons	*C*_*qp1*_, *C*_*qp2*_	*135*
Synaptic gain from pyramidal neurons to excitatory interneurons	*C*_*pq1*_, *C*_*pq2*_	*108*
Synaptic gain from slow inhibitory interneurons to pyramidal neurons	*C*_*sp1*_, *C*_*sp2*_	*33*.*75*
Synaptic gain from pyramidal neurons to slow inhibitory interneurons	*C*_*ps1*_, *C*_*ps2*_	*33*.*75*
Synaptic gain from fast inhibitory interneurons to pyramidal neurons	*C*_*fp1*_, *C*_*fp2*_	*40*.*5*
Synaptic gain from pyramidal neurons to fast inhibitory interneurons	*C*_*pf1*_, *C*_*pf2*_	*27*
Synaptic gain from fast inhibitory interneurons to slow inhibitory interneurons	*C*_*fs1*_, *C*_*fs2*_	*10*.*8*
Synaptic gain of fast inhibitory interneurons self-feedback	*C*_*ff1*_, *C*_*ff2*_	*135*
Noise excitation weight for pyramidal neurons	*K*_*p1*_, *K*_*p2*_	*40*
Noise excitation weight for fast inhibitory interneurons	*K*_*f1*_, *K*_*f2*_	*108*
Average time constant of pyramidal neurons membrane potential, the inverse divided by 2π is equivalent to low cut-off frequency [*sec*]	*ω*_*p1*_^*-1*^, *ω*_*p2*_^*-1*^	*10*^*−1*^
Average time constant of between node pyramidal neurons membrane potential, the inverse divided by 2π is equivalent to low cut-off frequency [*sec*]	*ω*_*p21*_^*-1*^, *ω*_*p12*_^*-1*^	*100*^*−1*^
Average time constant of excitatory interneurons membrane potential, the inverse divided by 2π is equivalent to low cut-off frequency [*sec*]	*ω*_*q1*_^*-1*^, *ω*_*q2*_^*-1*^	*100*^*−1*^
Average time constant of slow inhibitory interneurons membrane potential, the inverse divided by 2π is equivalent to low cut-off frequency [*sec*]	*ω*_*s1*_^*-1*^, *ω*_*s2*_^*-1*^	*50*^*−1*^
Average time constant of fast inhibitory interneurons membrane potential, the inverse divided by 2π is equivalent to low cut-off frequency [*sec*]	*ω*_*f1*_^*-1*^, *ω*_*f2*_^*-1*^	*200*^*−1*^
Average dendritic gains of pyramidal neurons [*mV*]	*G*_*p1*_, *G*_*p2*_	*0*.*32*
Average dendritic gains of between node pyramidal neurons [*mV*]	*G*_*p21*_, *G*_*p12*_	*3*.*2*
Average dendritic gains of excitatory interneurons [*mV*]	*G*_*q1*_, *G*_*q2*_	*3*.*2*
Average dendritic gains of slow inhibitory interneurons [*mV*]	*G*_*s1*_, *G*_*s2*_	*22*
Average dendritic gains of fast inhibitory interneurons [*mV*]	*G*_*f1*_, *G*_*f2*_	*50*
Expected spiking threshold voltage [*mV*]	*v*_*θ*_	*5*
Half-maximum firing rate [*Hz*]	*υ*_*0*_	*2*.*5*
Variance of membrane potential over individual neurons in the population *[mV*^*-1*^*]*	*R*	*1*.*12*
Variance of white noise *[Hz*^*2*^*]*	*σ*^*2*^_*1*_, *σ*^*2*^_*2*_	*0*.*5*
Between node synaptic gain	*K*_*21*_, *K*_*12*_	*40*
Average time constant of self-synaptic decay in fast inhibitory interneurons [*sec*]	*τ*_*f1*_	*0*.*01*
	*τ*_*f2*_	*0*.*005*

#### Noise sources and fast oscillations

Table 2 shows how the values of the main parameters *P*_*1*_ and *P*_*2*_ were systematically varied in order to enable different types of CFCs. Two independent noise sources, $N(P_{1},\sigma_{1}^{2})$ and $N(P_{2},\sigma_{2}^{2})$, excite both the pyramidal neurons and the fast inhibitory interneurons of Nodes 1 and 2, respectively. The noise sources represent the unmodeled mean firing rates, which originate from various cortical and subcortical regions. As long as the mean noise level *P* is too small, fast oscillations fail to arise due to damping. Higher levels of noise trigger fast oscillations, for which *P* serves as a bifurcation parameter. We will refer to the bifurcation value as limit-cycle threshold. When a *P* parameter value is below the limit-cycle threshold, the fast inhibitory population with feedback serves as a resonance filter. Such a filter is selective to a specific frequency in the input and the output contains the resonance frequency component. In this regime, the frequency of the fast oscillation is sensitive to the slow oscillations in the modulating input. Hence, the resonance is suitable as a frequency modulation mechanism. For PFC and FFC simulations, the parameter was set in the resonance range. When *P* value is above the threshold, a limit cycle emerges corresponding to the fast oscillation. In this type of oscillation, the amplitude of the fast oscillation is sensitive to modulatory input. Therefore, the limit cycle oscillation is a suitable mechanism for amplitude modulation. For PAC and AAC simulations, thus, the *P* parameters are set to obtain a limit-cycle osillation. For AFC, *P* values were set to obtain a limit cycle in one node and resonance oscillations in the other.

**Table 2:** *P* parameters for each CFC simulation.

Interpretation	Notation	cross-node PFC	cross-node PAC	FFC	AAC	AFC
Mean Input Noise Level	*P*_*1*_	*4*.*5*	*7*	*4*.*5*	*7*	*7*
	*P*_*2*_	*0*	*0*	*4*.*5*	*7*	*4*.*5*

Note: the value 0 deactivates fast oscillation; 4.5 induces a resonance regime and 7 induces a stable limit cycle corresponding to fast oscillations.

#### Slow oscillations, generation and synchronization

As in previous NMM studies [29,45], the model generates slow oscillations through the interactions between the pyramidal and slow inhibitory interneuron populations within each node. When nodes are connected, the slow oscillations synchronize, desynchronize, or show intermittent synchronization behavior, depending on the coupling strengths *K*_*12*_ and *K*_*21*_ between them [46]. Synchronization will not arise for small values of *K*_*12*_ and *K*_*21*_. We used *K*_*12*_ = *K*_*21*_ = 40 throughout this study, which assures that the slow oscillations are synchronized.

#### Choice of slow and fast oscillation frequencies

For frequency modulation, intuitively, the condition *f*_*fast*_ >> *f*_*slow*_ should be met where *f*_*fast*_ is the frequency of the fast oscillation and *f*_*slow*_ is that of the slow one. We set the slow oscillation in the delta band and the fast oscillation in the gamma band, the slowest and the fastest commonly observed in EEG. To generate slow oscillation in the delta band (~3Hz), the time constants of the pyramidal neurons in both nodes were set to $\omega_{p1}=\omega_{p2}=10\frac{rad}{s}$. This value differs by a factor 10 from that used in previous studies to obtain alpha oscillations [29,30,36]. In order to keep the stationary conditions of the model unaffected, the low frequency gain ($\frac{G}{\omega }$) was kept in the same proportion, meaning G was changed from 3.2 (for alpha) to 0.32 (for delta). The frequency of the fast oscillations is tuned through the time constant of the dynamic feedback, *τ*_*f*1_ and *τ*_*f*2_. These were set to 0.5 ms and 1ms, respectively, resulting in 51Hz and 42Hz gamma activity in the first and the second node, respectively. These frequencies were chosen to be different in order to show that for FFC, AAC, and AFC in general the fast oscillations do not need to have the same frequencies. For amplitude modulation, the condition should be met that $\frac{f_{fast}}{f_{slow}}>2$ [27]. The fast and slow frequencies chosen above meet these conditions.

### Evaluation of Cross-Frequency Coupling (CFC)

To assess the CFC generated by our model, the output of each node was decomposed into fast-oscillation (FO) and slow-oscillation (SO) signals, respectively, by high-pass or low-pass filtering at a 15Hz cut-off frequency (*f*_*c*_). For filtering we used the eeglab13.4.4b Matlab toolbox [47]. FO and SO signals were always normalized to zero mean and unit variance. To evaluate frequency modulation, the average number of zero crossings of the FO signal was calculated for each positive and negative phase of the SO signal in the same node. The average number of zero crossings was obtained by calculating the zero-crossing rate according to the following equation:

$$zcr=\frac{1}{2T}\sum_{t=0}^{T\times f_{s}}\text{I}\{FO(t)\times FO(t+1)<0\},$$

in which *T* is the time interval of SO in negative (from sine(-π) to sine(0)) or positive (from sine(0) to sine(π)) phases and *f*_*s*_ is the sampling frequency. The indicator function I{*A*} is 1 if its argument is true and 0 otherwise. The average over *T* does not reflect small fluctuations, which do not cross zero. As a result, the number *zcr* tends to be lower than the actual frequency, i.e., the average numer of zero crossing is *not* identical to the average frequency, but may serve as an index is of it. The power spectral density (PSD) of the output signal was calculated using the [48] method. A Hanning time window of length equal to 16348 data point was moved stepwise with 25% overlap. Amplitude modulation was evaluated by visual inspection, comparing the envelope of the FO signals with the SO signal.

### Simulation specifications

The model was implemented and ran in Matlab/Simulink 8.0 (R2012b), using a 4^th^ order Runge—Kutta method with fixed time step of 0.0001 seconds. The number of iterations was chosen sufficiently large to assure stationarity of the dynamics. Transient dynamics were omitted from data analyses and graphical presentations of the results. The model is available at Zenodo website [49].

## Results

### Phase-frequency coupling

**Fig 2:**
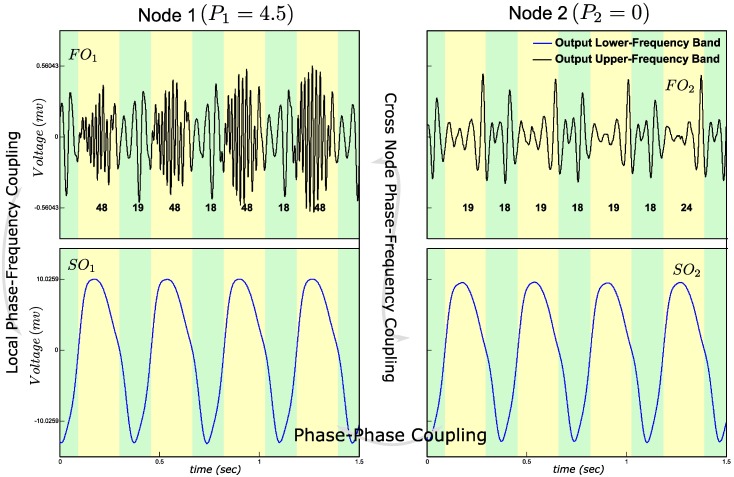
Phase-Frequency Coupling (PFC) simulation. Time domain signals are shown for visual inspection of each CFC. FO_1_, SO_1_, FO_2_ and SO_2_ are the filtered output of the Node 1 and Node 2 respectively. The frequency of FO_1_ varies between positive (yellow stripes) and negative (green stripes) phases of SO_1_. SO_1_ and SO_2_ are synchronized (i.e., cross-node PPC). Integers below the FO time waves are average numbers of FO zero crossings, which is used as an index of average frequency of each phase. Local PFC between FO_1_ and SO_1_ and cross-node PFC between FO_1_ and SO_2_ are observed.

For *P*_*1*_ a value of 4.5, lower than the limit cycle threshold, was selected (Table 2), which assures that the node operates as a resonance filter circuit in the gamma band. As shown in Fig 2, for every positive phase of the slow oscillation in Node 1, the average zero crossings of fast oscillation in Node 1 was increased and for every negative phase it was decreased. Amplitude of the fast oscillation was slightly modulated as well, but far less pronounced than frequency. This illustrates that in the resonance regime, the fast oscillation is more sensitive to frequency than to amplitude modulation. The value of *P*_*2*_ was set to 0 in order to deactivate the fast oscillation in the second node. The PFC arises within Node 1. The slow oscillation in the first node was synchronized with that in the second node (Fig 2). As a consequence, the frequency of the fast oscillation in Node 1 also correlates with the phase of the slow oscillations in Node 2, resulting in cross-node PFC. Note that cross-node PFC was generated only through the settings of mean noise input levels, *P*_*1*_ and *P*_*2*_, while connection strengths remained fixed. The cross-node PFC arises through cross-node phase-phase coupling of the slow oscillations, combined with local phase-frequency coupling. Although the fast oscillation in Node 2 appears to be oscillating at around 18Hz, this is due to the cut-off frequency of the filters. As we will report in the *Power spectrum densities (PSDs) of Model output*, the inhibitory interneurons in Node 2 do not produce any fast oscillations.

### Phase-amplitude coupling

**Fig 3:**
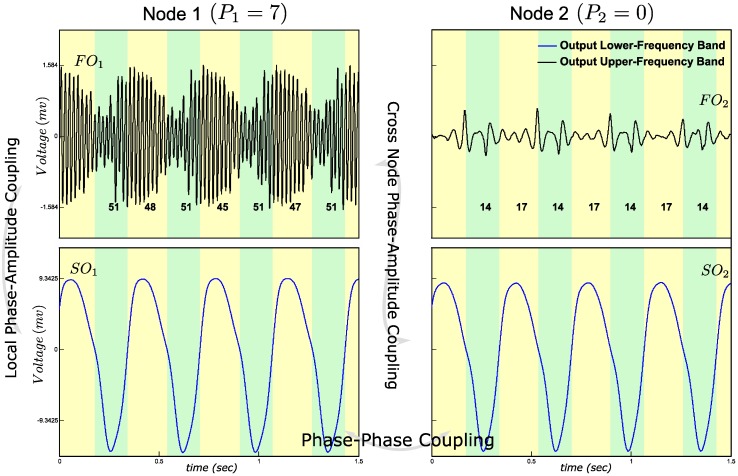
Phase-Amplitude Coupling (PAC) simulation. The instantaneous amplitude of FO_1_ correlates with the synchronized SO_1_ and SO_2_. Local PAC between FO_1_ and SO_1_ and cross-node PAC between FO_1_ and SO_2_ are observable. Integers below the FO time waves indicate average number of zero crossings of FOs.

The mechanism for implementing cross-node PAC is similar to above, except that in the first node, the value of parameter *P*_*1*_ was set to 7, which is higher than the limit cycle threshold which triggers a steady-state gamma band oscillation. *P*_*2*_ again was set to 0, in order to deactivate the fast oscillation in the second node (Table 2). The amplitude of the fast oscillation in Node 1 was modulated by the slow activity via the coupling between the slow and the fast inhibtory interneurons. Through the synchronization of the slow oscillations across the nodes, the slow oscillation in Node 2 also correlates with the amplitude changes of the fast oscillation in Node 1, resulting in cross-node PAC (Fig 3).

### Frequency-frequency coupling

**Fig 4:**
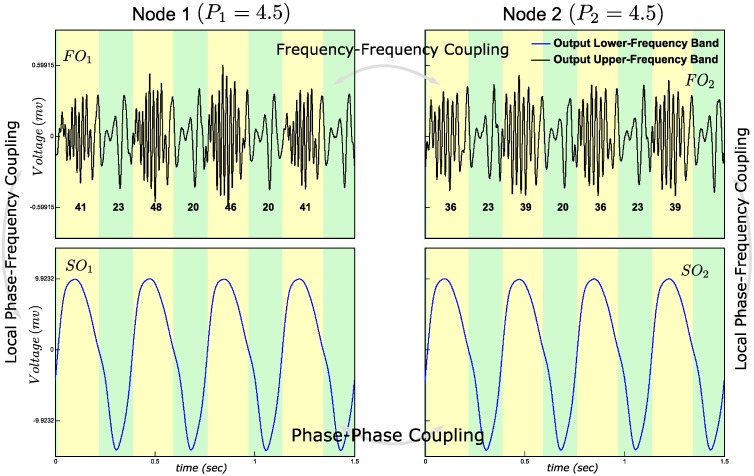
Frequency-Frequency Coupling (FFC) simulation. The frequency of FO_1_ and FO_2_ varies for the synchronized SO_1_ and SO_2_. Integers below the FO time waves are the average number of zero crossings, which is an index of average frequency of each phase. The numbers change between positive and negative phases of synchronized SO_1_ and SO_2_. Local PFC between FO_1_ and SO_1_, and between FO_2_ and SO_2_ are also observed.

With FFC, two distinct fast oscillations change their frequencies over time. Thus, two frequency-modulated signals are required. To enable this, *P*_*1*_ and *P*_*2*_ were both set to 4.5, i.e. in the resonance regime. The resulting FFC is shown in Fig 4. Fast oscillations in both nodes show increase and decrease in average number of zero crossings within, respectively, the negative and positive phases of the slow oscillations. The synchronized slow oscillations modulate the frequency of the fast oscillations. Thus, the FFC is the result of two local PFCs of which the slow signals are synchronized between nodes.

### Amplitude-amplitude coupling

**Fig 5:**
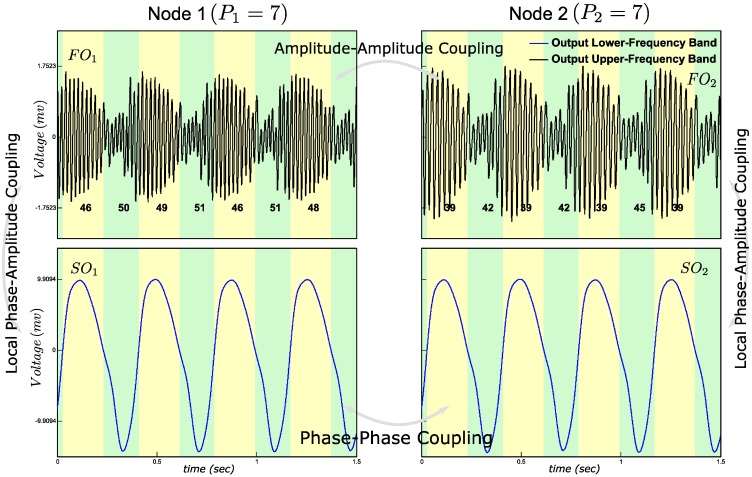
Amplitude-Amplitude Coupling (AAC) simulation. The instantaneous amplitude of FO_1_ and FO_2_ correlate with the synchronized SO_1_ and SO_2_. Integers below the FO time waves indicate average number of zero crossings of FOs.

AAC refers to the correlation between two amplitude signals from two distinct fast oscillations. Therefore, AAC requires a pair of amplitude-modulated signals. To achieve this, *P*_*1*_ and *P*_*2*_ were both set to 7, well above the limit cycle threshold. Time constants for the fast interneuron populations self-feedback were set to obtain a fast oscillation of about 51Hz in Node 1, and of about 42Hz in Node 2. As shown in Fig 5, the amplitudes of the fast oscillations were modulated by synchronization between the slow oscillations. As a result, the amplitude of the fast oscillation in Node 1 correlated with that of the fast oscillation in Node 2. AAC is thus the product of two local PACs of which the slow signals are synchronized between nodes.

### Amplitude-frequency coupling

**Fig 6:**
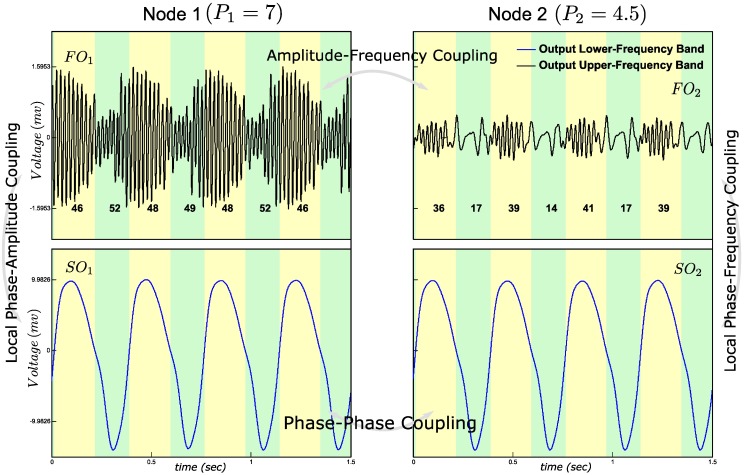
Amplitude-Frequency Coupling (AFC) simulation. The instantaneous amplitude of FO_1_ correlates with the synchronized SO_1_ and SO_2_. The frequency of FO_2_ is about constant while the frequency of FO_1_ varies for the synchronized SO_1_ and SO_2_. See average number of zero crossings in FOs, indicated as integers below the FO time waves.

AFC is the most complex type of CFC; amplitude of one fast oscillation correlates with the fluctuations in the frequency of the other. To obtain AFC, PAC is generated in Node 1, i.e., the *P*_*1*_ is set to 7, well above the limit-cycle threshold value, and *P*_*2*_ is set to 4.5, in the resonance regime. PAC within Node 1 and PFC within Node 2 were combined via the cross-node phase synchronization. The synchronized slow oscillations between nodes simultaneously modulate the amplitude of the fast oscillation in Node 1 and the frequency of the fast oscillation in Node 2, resulting in AFC between the fast oscillations of Nodes 1 and 2 (Fig 6).

### Power Spectrum Densities (PSDs) of model output

**Fig 7:**
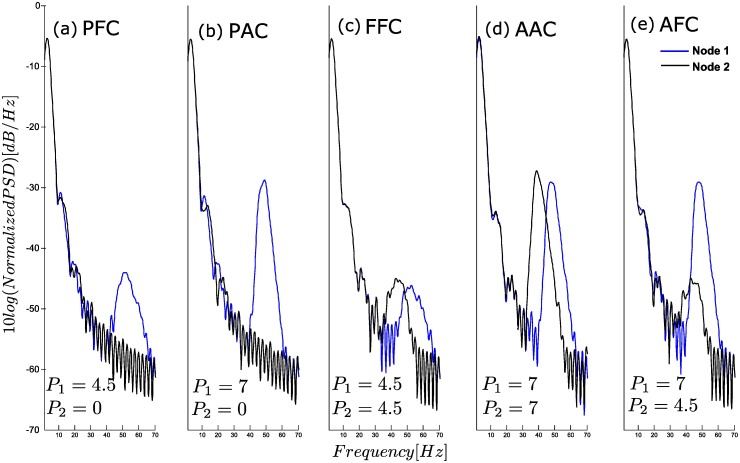
The comparison of PSDs in different forms of CFC. PFC: Phase-Frequency Coupling, PAC: Phase-Amplitude Coupling, FFC: Frequency-Frequency Coupling, AAC: Amplitude-Amplitude Coupling, AFC: Amplitude-Frequency Coupling.

Fig 7 shows PSDs of outputs from Nodes 1 and 2 in each CFC simulation. Peaks appear in the delta (< 4Hz) and gamma (>30Hz) bands, which indicates stationary oscillatory activity. In case of frequency modulation (Panels a and c), the gamma activity shows wider peaks than in case of amplitude modulation (Panels b and d). In Panel e, where both modulations occur, a narrow (Node 1) and a wide (Node 2) bands can be observed in the gamma activity. In the PFC and PAC results (Figs 2 and 3), the fast oscillation in Node 2 appeared to have oscillatory activity around 18Hz. However, no peak at 18Hz exists in Panels a and b. Thus, the apparent ‘18Hz oscillation’ is shown to be an artifact of filtering. (Note that numbers of zero crossings of fast oscillations in Figs 2–6 are slightly lower than that of their actual frequency due to the estimation method of the zero crossing)

## Discussion

As suggested by Jensen and Colgin [2], coupling between slow and fast neural activity could take one of six forms: PFC, PAC, FFC, AAC, AFC or PPC. The first five of these were obtained in a model consisting of two mutually connected four-populations NMM nodes. For PAC or PPC, models have previously been developed with greater neurological detail than the present one [50–52]. Instead, we aimed to understand the systematic relationship among the cross-frequency couplings (CFCs). Neural mass models afford this based on their relative simplicity while preserving sufficient neural plausibility for simulating population level electrophysiological signals [30,33,45]. The neural mass modeling approach has been used for both quantitative estimation of the system parameters in cortical and subcortical regions [43,53–55] and qualitative simulation for neural systems behavior [31,56–58]. Here, all results are qualitative, as they serve the purpose of introducing a plausible common mechanism for the emergence of different types of CFC.

Each node of the model represents a patch of cortical tissue, anywhere between a few millimeters and a centimeter in diameter, in which oscillations in various frequencies can be observed [41]. Whereas nodes were connected symmetrically to represent interconnected brain electrical activity sources within a cortical region, asymmetric inter-node connectivity is more likely to represent hierarchical structures, such as top-down/bottom-up connectivity in visual cortex [59]. Addressing this issue will require further elaboration of the inter-node connectivity of the model.

To generate different types of CFC, the model structure and connectivity were not changed, rather, the level of input noise of the nodes (parameters *P*_*1*_ and *P*_*2*_) was varied. The noise level determines the stability of the fast activity: when *P* is in the resonance regime, local PFC occurs, while the parameter is set above the limit-cycle threshold, local PAC emerges. The results showed that PFC and PAC are two phases of a CFC which are determined by the stability of the fast inhibitory activity. The modeled fast activity represents interneuron gamma (ING). Thus, the simulation results predict that, as the mean noise input level to a cortical region changes, stability of ING will change, and CFC will change in appearance between frequency and amplitude modulation.

The slow activity of the nodes was synchronized via the inter-node connection. The local fast activities are modulated via the synchronized slow activity. Our results show how local ING can be modulated across brain regions via a common intrinsic slow activity, e.g., alpha band activity in visual cortices. Cross-regional PAC was reported even between bilateral motor cortices in monkeys [60]. For the inter-hemispheric PAC, other regions, such as, the subthalamic nucleus, could also contribute as a pacemaker. A pacemaker region could replace or complement the between-node pyramidal population [43,61]. This would not affect to the framework of cross-regional PAC/PFC generation, as long as the slow activities of the individual nodes remain synchronized in the same frequency band. Across brain regions of which the main frequencies differ, e.g., alpha rhythms in visual cortices and theta in hippocampus, m:n synchrony (where m≠n) is needed for modulation of local fast activities. Neural mass modeling studies have been addressing the issue of cross-frequency phase-phase coupling (PPC), in which coupling strength between the nodes is the major parameter of interest [46]. The current study, however, does not address the issue since our main parameters were the mean noise input levels.

The model also successfully implemented AAC, FFC and AFC. In all simulations, amplitude and/or frequency of fast activity was modulated by synchronized slow activity. The assumption of a common modulator is in accordance with empirical studies. For example, AAC measurement provided evidence that two fast activities were modulated by a common slow activity in LFP signals [24–26]. To our knowledge, PFC, FFC and AFC have not yet been reported in brain signals. The model predicts FFC if slow activity in two regions is phase synchronized while stability of ING in both is intermediate, i.e., in the resonance regime. Likewise, AFC is expected when the slow activity is synchronized while ING is stable in one region but intermediately stable in the other.

Our PFC, FFC and AFC results provide a possible explanation why frequency modulations are scarcely reported. Frequency modulation occurred when the model was in the resonance regime, where amplitude modulation also occurrs to some extent. This suggests that in the brain signal, PFC and PAC may co-exist. Since PFC measures are still under-developed compared to PAC measures [6,62–65], PFC may fail to be observed in a noisy signal such as EEG, while the weaker PAC is detected.

The frequency modulation could be more than a mere transient state to amplitude modulation. Empirical studies have shown that fast inhibitory interneurons fire at a specific phase of the gamma band oscillation [37,66]. The gamma frequency, in this context, is synonymous to the average number of spiking. As the gamma frequency increases/decreases according to the slow phases, the inhibitory population changes in excitability. In the current model, the range between the maximum and minimum frequencies is larger in the frequency modulation than in the amplitude modulation. Such a wide range of excitability is beneficial, for example, for implementing a gating function for information transfer.

In our model, frequency was determined by the fixed time constants of the neural populations (see also [67]). Frequency modulation was realized by through the effect of mean input noise level on the gain of the sigmoid block. The product of the gain and the time constant determines the resonance frequency. In principle, the time constants could also be considered as function of noise level [40]. This, however, would not change the principle behind the current approach.

We conclude that two fundamental forms of CFCs in cortex are PFC and PAC. They emerge as modulations of ING. The couplings can either be kept local or spread across regions via PPC, depending on the synchronization of slow intrinsic oscillation between these regions.
